# Multi‐country outbreak of *Salmonella* Bovismorbificans ST377 infections linked to the consumption of alfalfa sprouted seeds 25 June 2026

**DOI:** 10.2903/j.efsa.2026.10220

**Published:** 2026-07-14

## Abstract

Between January and May 2026, 109 confirmed cases of *Salmonella* Bovismorbificans ST377 were reported from 10 European Union/European Economic Area (EU/EEA) countries (Austria, Belgium, Czechia, Denmark, Finland, Germany, Ireland, Luxembourg, Spain and the Netherlands) and the United Kingdom. The outbreak predominantly affected adult females. Eighteen cases required hospitalisation. Two deaths were reported in Finland (one confirmed and one probable case).

Epidemiological and microbiological evidence identified alfalfa sprouted seeds traded from Italy as the primary vehicle of infection. Microbiological evidence included the detection of the outbreak strain in water samples collected during alfalfa sprouted seed harvesting in the Netherlands and Northern Ireland (UK). A Finnish sprouted seed producer was epidemiologically linked to cases in Finland.

Traceability investigations in Italy identified a common seed supplier in India, suggesting this area as a potential origin of seed contamination. The outbreak strain presumably started circulating in Europe in October 2025 via two alfalfa seed consignments, before being distributed across multiple countries.

Control measures included the withdrawal of the implicated consignments, recalls of related products, cessation of production and the destruction of suspected products. Following these interventions, case notifications decreased. However, further infections may occur until the source of contamination is fully identified and controlled, particularly because sprouted seeds can be sold as ready‐to‐eat products, representing a concern for microbial food safety.

Based on available information, the risk of infections is assessed as low‐to‐moderate for people in EU/EEA countries who frequently consume sprouted seeds.

Public health authorities are encouraged to interview new cases, sequence isolates, and share information in EpiPulse. Food safety authorities are encouraged to investigate the role of the environment in seed contamination. Seed producers should implement appropriate measures to minimise the contamination risk. Sprouted seed producers should implement adequate food safety management systems to ensure safe products reach the market.

Suggested citation: European Centre for Disease Prevention and Control, European Food Safety Authority, 2026. Multi-country outbreak of *Salmonella* Bovismorbificans ST377 linked to the consumption of alfalfa sprouted seeds – 25 June 2026. Catalogue number: TQ-01-26-046-EN-N, ISBN: 978-92-9498-901-7, doi: 10.2900/2128081.

Also published in EFSA Journal: Scientific report approved by EFSA on 25 June 2026; doi:10.2903/j.efsa.2026.10220; Key words: *Salmonella*, sprouted seeds, multi-country outbreak, Whole Genome Sequencing (WGS). Requestor: European Commission; Question number: EFSA-Q-2026-00272; correspondence: Ask a question, ISSN: 1831-4732.

## Event background

On 16 April 2026, Ireland reported three cases of *Salmonella* Bovismorbificans to the European surveillance portal for infectious diseases (EpiPulse events, 2026-FWD-00040, outbreak strain accession number ERR16978937). The cases were indistinguishable at core genome multilocus sequence typing (cgMLST) level, with an epidemiological and microbiological link to the consumption of sprouted seeds.

After the alert from Ireland, other European countries reported additional cases clustering with the Irish strain, and this confirmed the multi-country spread of the specific strain of *S.* Bovismorbificans.

Sprouted seeds have been associated with outbreaks of salmonellosis before. On 6 March 2025, the European Centre for Disease Prevention and Control (ECDC) and the European Food Safety Authority (EFSA) published a joint rapid outbreak assessment 'Prolonged cross-border multi-serovar *Salmonella* outbreak linked to consumption of sprouted seeds' [1]. The outbreak involved 509 reported cases of salmonellosis during the period 2023–2025, and the *Salmonella* serovars identified included Adelaide, Enteritidis, Hvittingfoss, Kinondoni, Kisarawe, Newport, Richmond, and two distinct strains of Typhimurium.

On 11 May 2026, ECDC and EFSA initiated a joint rapid outbreak assessment as a follow-up to the outbreak of *S.* Bovismorbificans, recognising that outbreaks of *Salmonella* linked to sprouted seeds have previously occurred between 2023 and 2025. Due to an ongoing risk of infections with *S.* Bovismorbificans linked to consumption of sprouted seeds in a number of EU/EEA countries, this rapid outbreak assessment aims to trigger further public health and food safety investigations.

### Outbreak strain characterisation

The *S.* Bovismorbificans outbreak strain belongs to sequence type (ST) 377 and contains very few antimicrobial resistance markers, with some isolates containing none (analysed using the Resfinder tool).

### European outbreak case definition

The European outbreak case definition for a **confirmed outbreak case** is as follows:

A laboratory-confirmed *Salmonella enterica* serovar Bovismorbificans case with symptom onset on or after 1 January 2026 (date of sampling or date of receipt by the reference laboratory if date of onset is not available);

AND

fulfilling at least one of the following laboratory criteria for an *S.* Bovismorbificans ST377 isolate sequence:

- clustering in the national cgMLST pipeline within five core genome (cg) allelic differences (alternatively five Single Nucleotide Polymorphisms) from any of the two representative outbreak strains ERR16978937 and ERR16945376.
- clustering within five cg-allelic differences in single-linkage analysis using the ECDC's centralised Molecular Typing Tool.

OR

fulfilling the epidemiological criterion:

- epidemiologically linked to a confirmed case, based on a national outbreak case definition.

The European outbreak case definition for a **probable outbreak case** is as follows:

A laboratory-confirmed *Salmonella enterica* serovar Bovismorbificans case with symptom onset on or after 1 January 2026 (date of sampling or date of receipt by the reference laboratory if date of onset is not available).

## Epidemiological and microbiological investigations of human cases

As of 16 June 2026, 11 European countries reported 109 confirmed cases of *S.* Bovismorbificans ST377 linked to the outbreak. The highest numbers were observed in the United Kingdom (30), Finland (35), and the Netherlands (17). Additional confirmed cases were reported by Austria (2), Belgium (4), Czechia (1), Denmark (4), Germany (6), Ireland (6), Luxembourg (3) and Spain (1). Four countries also reported ongoing investigations of additional probable cases: Finland (30), Germany (13), Poland (4) and Spain (3).

Most cases were adults, with some children affected, and a majority were female in four countries. Hospitalisation was reported in nine countries, including Denmark (2), Finland (six confirmed cases and four probable cases), Germany (1), Ireland (2), Luxembourg (2), Poland (four probable cases), the Netherlands (2), Spain (1), and the United Kingdom (2). The case reported by Spain involved a Finnish tourist visiting Spain, no other travel-related cases were reported.

Two deaths were reported in Finland, one in a confirmed case and one in a probable case, occurring within 0–30 days after infection. Both cases had underlying health conditions possibly related to the death, and one of the cases was receiving terminal care. The overall description of the demographic information is detailed in Table 1.

**Table 1. Demographic summary of confirmed *Salmonella* Bovismorbificans outbreak cases, stratified by country in 10 EU/EEA countries and the United Kingdom**

| Country | No. of confirmed cases | Female | Male | Disease onset (range)<br>All dates are from 2026 | Median (years) Age (range) | No. of hospitalised cases |
|---|---|---|---|---|---|---|
| Austria | 2 | 2 | - | 10 April – 16 May | 50 (31–69) | - |
| Belgium | 4 | 3 | - | 1 April – 22 April | 66 (*) | - |
| Czechia | 1 | 1 | - | - | 20 | - |
| Denmark | 4 | 2 | 2 | 4 April – 6 April | 53 (25–67) | 2 |
| Finland | 35 | 26 | 9 | 6 March – 15 May (unclear onset date for the index case, sample date given) | 44 (4–77) | 6 |
| Germany | 6 | 2 | 4 | 26 March – 27 April | 65 (43–77) | 1 |
| Ireland | 6 | 4 | 2 | 15 January – 7 May | 52 (19–77) | 2 |
| Luxembourg | 3 | 3 | - | 13 April – 11 May | * (40–88) | 2 |
| Spain | 1 | - | 1 | 29 March | 14 | 1 |
| Netherlands | 17 | 13 | 4 | 20 February – 7 May | 58 (15–87) | 2 |
| UK | 30 | 21 | 9 | 14 January – 8 May (sample dates) | 54 (17–77) | 2 |
| **Total for EU/EEA** | **79** | **56** | **22** | **15 January – 16 May** | ***(4–88)** | **16** |
| **Total for Europe (EU/EEA and UK)** | **109** | **77** | **31** | **14 January – 16 May** | ***(4– 88)** | **18** |

**not reported*

### Information from patient interviews

Epidemiological investigations indicated that a substantial number of interviewed cases in Finland (31), Ireland (4), the Netherlands (6), and the United Kingdom (12) reported consuming sprouted seeds, including some linked to a recalled brand. In a few cases, consumption of sprouted seeds could not be confirmed, although salads or bread containing seeds were reported. Overall, the findings support a link between the outbreak and the consumption of sprouted seeds. Eight countries provided interview data on sprouted seed consumption:

**Belgium** reported that both interviewed cases had consumed bread containing seeds, but neither recalled consuming sprouted seeds. The two other cases were not interviewed due to the prolonged time between diagnosis and cluster confirmation (>1 month).

**Denmark** reported that the two interviewed cases did not report consuming sprouted seeds.

**Finland** has interviewed a total of 49 cases; 31 reported consuming sprouted seeds, including both mung beans (10) and alfalfa (11), while 11 cases reported consuming sprouted seeds at a restaurant or were unable to identify the specific type consumed. Thirteen cases reported consumption of a specific local sprout brand. Further case interviews associated with restaurant visits (mainly buffets) identified 12 additional cases with probable consumption of the same brand. During the interviews, two other sprout brands were each mentioned once.

**Germany** reported that two interviewed cases could not recall consuming sprouted seeds, and one additional case was hospitalised in critical condition and could not be interviewed.

**Ireland** reported that four cases reported consuming sprouted seeds, including two who had consumed a product containing a mix of alfalfa, radish, clover, and broccoli seeds. The other two cases were unable to recall knowingly consuming sprouted seeds; one of these cases had travelled to the Netherlands prior to symptoms onset. Two cases (>65 years of age) were hospitalised due to infection.

**Luxembourg** has interviewed three cases. One case was a resident of a long-term care facility where sprouted seeds were served. Two cases did not recall consuming sprouted seeds; one reported following a vegan diet, while the other reported regularly consuming pre-packed salads.

**The Netherlands** has interviewed eleven cases, six reported consuming sprouted seeds, including mung beans (3) and alfalfa (3).

**The United Kingdom** has interviewed 14 cases; 12 reported consuming sprouted seeds. Six of the twelve cases reported consumption of a recalled brand, while one could not recall the specific brand. Two additional cases reported consuming alfalfa sprouts. The remaining two cases did not report consuming sprouted seeds but did report consuming salad or other salad-related items.

## Microbiological and environmental investigations of food and control measures

This section summarises the results of the food investigations carried out by the food safety authorities as part of this multi-country outbreak. The traceability and analytical results were those shared by the countries involved under the Rapid Alert System for Food and Feed (RASFF) Alert Notification 2026.3378 (51 European Commission (EC) validated follow-ups, *fups*) as of 29 May 2026. Figure 1 and Figure 2 display a graphical representation of the food traceability investigations related to the food vehicles suspected in this multi-country outbreak, namely alfalfa seeds from Batch A and Batch B and related sprouted seed products. The overall description of the traceability and microbiological information is detailed in Annex 1.

On 20 April 2026, the European Commission issued an RASFF notification (2026.3378) on behalf of United Kingdom (Northern Ireland) to inform about the detection of *S.* Bovismorbificans in a sample of water collected during an own check control at the sprouted seed Producer A located in Northern Ireland. Further investigation confirmed a microbiological link between the water irrigation isolate and human isolates from eight cases of infection (January–March 2026) initially investigated. The water sample (irrigation water from sprouted seeds, planted on 12 March 2026) was collected on 14 March 2026 (Figures 1 and 2).

In reply to this alert, the food safety authority in Finland communicated the findings of an ongoing national investigation (*fup1*, 2026.3378) in RASFF. One Finnish producer of sprouted seeds (Producer B) had been linked epidemiologically to some of the investigated cases (Figures 1 and 2).

On 21 May 2026, the food safety authority in the Netherlands reported the detection of *S.* Bovismorbificans matching the representative outbreak strain through RASFF. The isolate derived from a sample of rinsing water collected during an own check control at the sprouted seeds Producer F. The water sample tested positive on 14 February 2026 (*fup43*, 2026.3378). The harvest of sprouted seeds associated with the positive rinsing water sample was obtained using alfalfa seeds from Batch AS (*fup43*, *fup49*, 2026.3378) (Figure 2).

Producer A in the United Kingdom (Northern Ireland), Producer B in Finland, and Producer F in the Netherlands had been supplied with alfalfa seeds for sprouting by the same seed supplier, namely the Italian seed Supplier A.

In response to the ongoing national investigations, the food safety authority in Italy traced back and forward the batches of seeds (various typologies) that the Italian seed Supplier A had delivered to these three sprouted seed producers, and beyond. In particular, the tracing analysis focused on alfalfa seeds, which were considered to be the suspected vehicle as they had been sprouted concurrently with the detection of *S.* Bovismorbificans in the water samples in the United Kingdom (Northern Ireland) and the Netherlands, or were available at the time of the case investigations (Finland). The traceback analysis revealed that the batches of alfalfa seeds suspected in the United Kingdom (Northern Ireland) (Batch C), in Finland (Batch AJ), and in the Netherlands (Batch AS) originated from two alfalfa seed batches (Batch A (Figure 1) and Batch B (Figure 2)) purchased from India (Indian Supplier C), which were presumed to have arrived in Italy on 10 August 2025. The Italian seed Supplier A split these two consignments, assigned new batch numbers, and distributed them to multiple countries in Europe (13 EU/EEA, Switzerland, and the United Kingdom) and outside Europe.

As of 22 May 2026, the food safety authority in Italy informed that the Italian seed Supplier A had been asked to implement withdrawal procedures for the alfalfa seeds from Batch A and Batch B.

Meanwhile, the food safety authorities in the countries concerned by the distribution of the suspected seeds from Italian seed Supplier A have reported the implementation of control measures. In the United Kingdom (Northern Ireland), Producer A has ceased production while investigations are ongoing. In Finland, Producer B stopped using seeds from Batch AJ in late April and the products that were still on the market were withdrawn. In late May, sprouted seed products from alfalfa seeds Batch BW were recalled. In the Netherlands, the harvest of sprouted seeds associated with the positive result (positive rinsing water sample) was destroyed and not traded. This harvest was obtained with alfalfa seeds from Batch AS (*fup43*, *fup49*, 2026.3378) and was not marketed, but rejected and destroyed.

**Figure 1 and 2. Graphical representations of the traceability and microbiological investigations related to alfalfa seeds from Batch A and Batch B, as reported to RASFF by the countries involved (as of 29 May 2026)**

**Figure 1 (Batch A)**

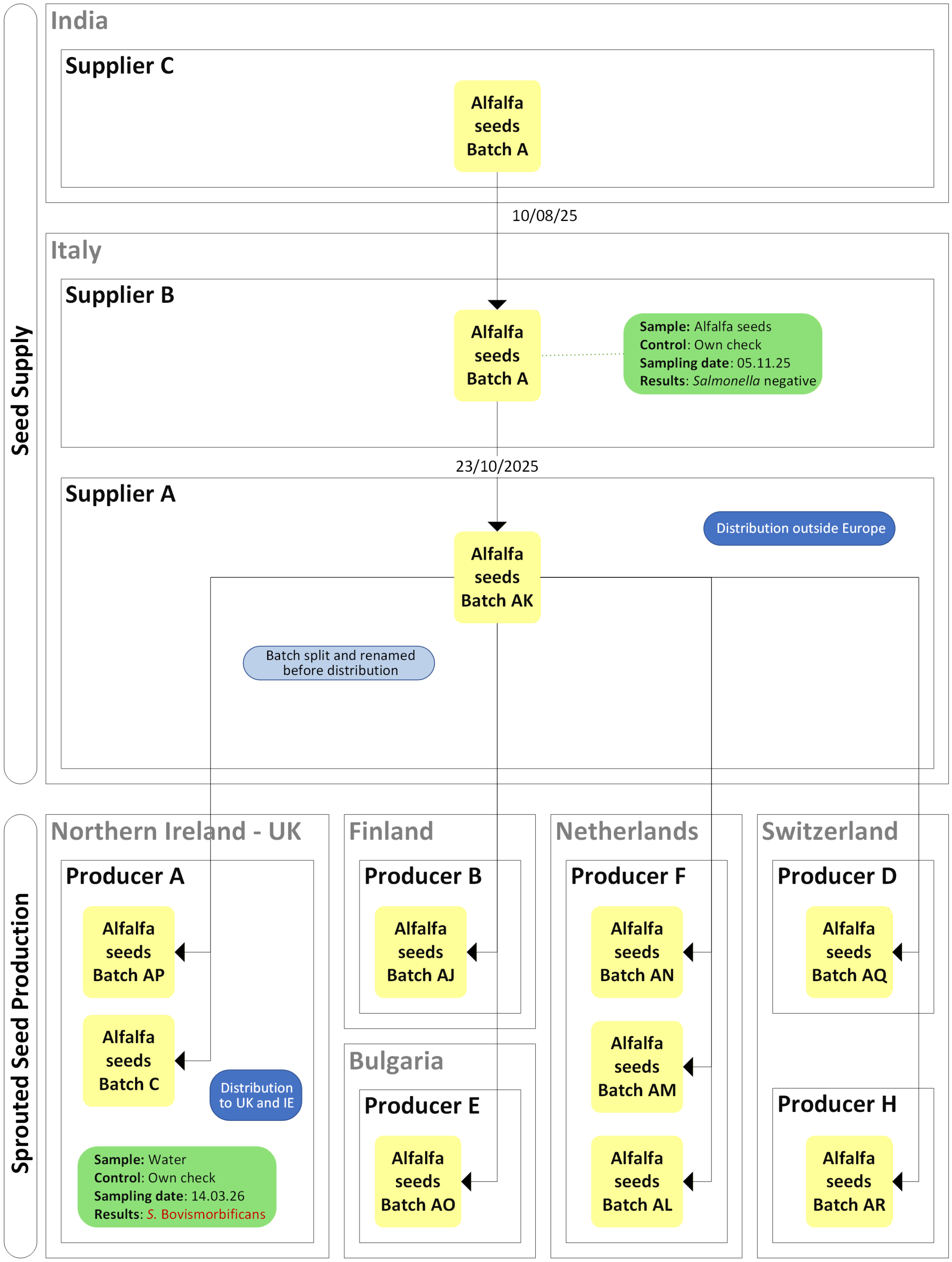

*Green boxes indicate sampling. Text in red in green boxes indicates* Salmonella *Bovismorbificans findings (outbreak strain). Yellow boxes indicate the products. Blue boxes indicate additional information. Arrows indicate trace forward distribution. Boxes refer to the food business operators and countries concerned.*

**Figure 2 (Batch B)**

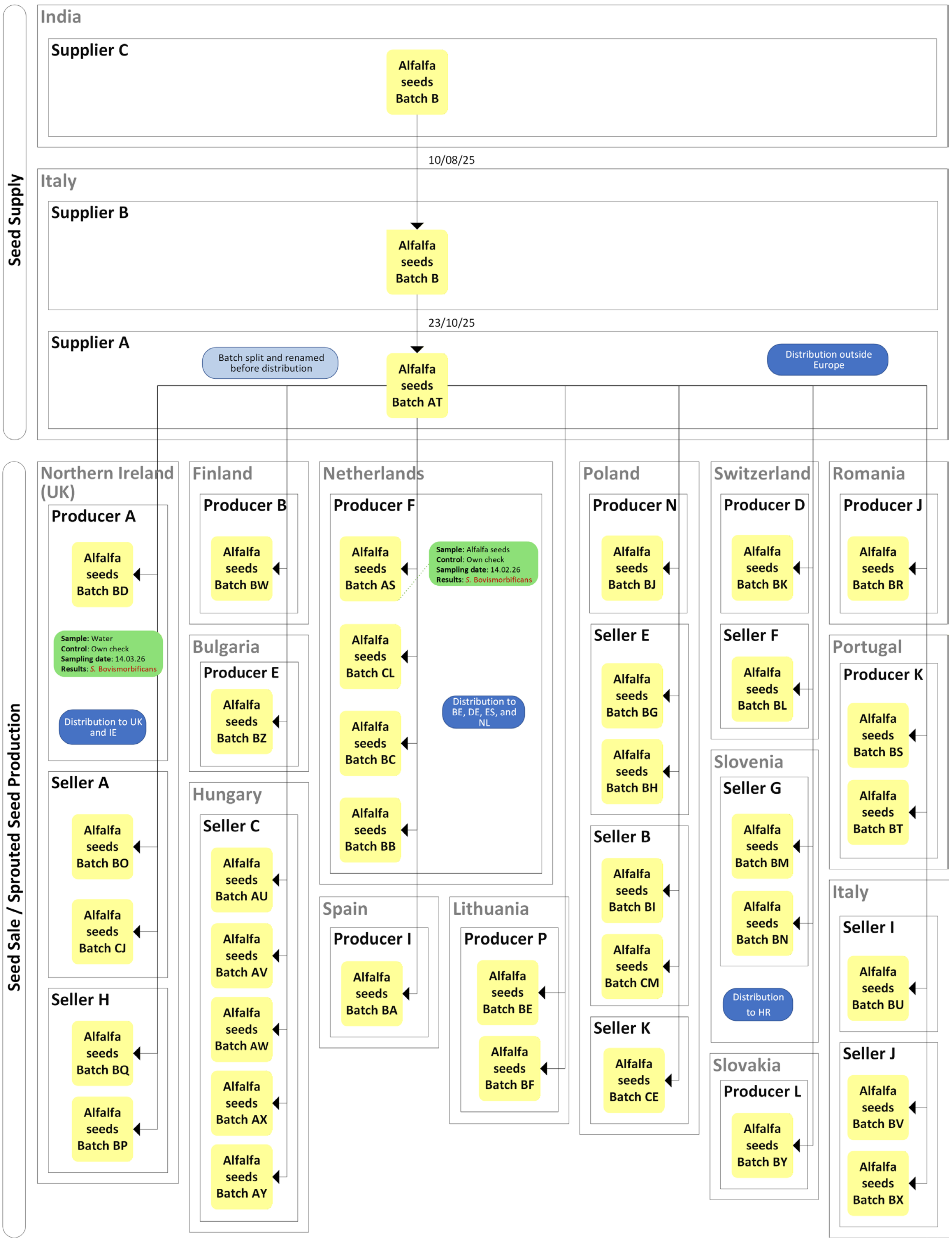

*Green boxes indicate sampling. Text in red in green boxes indicates* Salmonella *Bovismorbificans findings (outbreak strain). Yellow boxes indicate the products. Blue boxes indicate additional information. Arrows indicate trace forward distribution. Boxes refer to the food business operators and countries concerned.*

## European whole genome sequencing analysis of human and non-human isolates

### Whole genome sequencing data collection and cross-sectoral analysis

#### Human isolates

Cluster analysis was performed on isolate sequences shared in connection with the launched event and possible matching isolates in ECDC's whole genome sequencing (WGS) database. In total, 56 representative sequences were shared from seven countries, Czechia (1), Germany (2), Finland (2), Ireland (6), Luxembourg (3), the Netherlands (15), and the United Kingdom (27). All isolates were collected in 2026.

#### Non-human isolates

EFSA launched a call for data in May 2026. Countries were invited to submit genomic information on isolates of *S.* Bovismorbificans ST377, collected during the period 2025–2026 from seeds and sprouted seeds and matching the representative outbreak strains, to the EFSA WGS System. Seven countries (Austria, Finland, France, Iceland, Ireland, Norway and Poland) replied that they did not have non-human isolates with such characteristics.

#### Cross-sectoral analysis

For cross-sectoral analysis, the cgMLST analysis was performed at both ECDC and EFSA, as previously described [2]. Genome profiles were calculated from assembled genomes using chewBBACA version ≥ 2.8.5, according to the schema described by Rossi et al. 2018 [3] for *Salmonella* enterica, made available by Chewie Nomenclature Server [4]. Isolates with more than 10% of missing loci (325 from a total of 3 255 loci) were excluded from the analysis.

#### Results of cross-sectoral WGS analysis

ECDC queried the EFSA WGS system on 15 June 2026, using the sequences of the representative outbreak strains and European case definition allelic distances as thresholds in the query. In total, two non-human isolates matched the representative human isolates sequences (Figure 3). The two non-human isolates matching the representative outbreak strains are listed below.

- One isolate from an own check control sample of rinsing water from a harvest of sprouted alfalfa seeds, collected on the premises of Producer F in the Netherlands in February 2026.
- One isolate from an own check control sample of irrigation water (irrigation water from sprouted seeds) collected on the premises of Producer A in the United Kingdom (Northern Ireland) in March 2026.

**Figure 3. Single-linkage cluster tree of 56 human and two non-human *S.* Bovismorbificans ST377 isolate sequences collected in 2026, by country (Czechia, Germany, Finland, Ireland, Luxembourg, Netherlands, and UK)**.

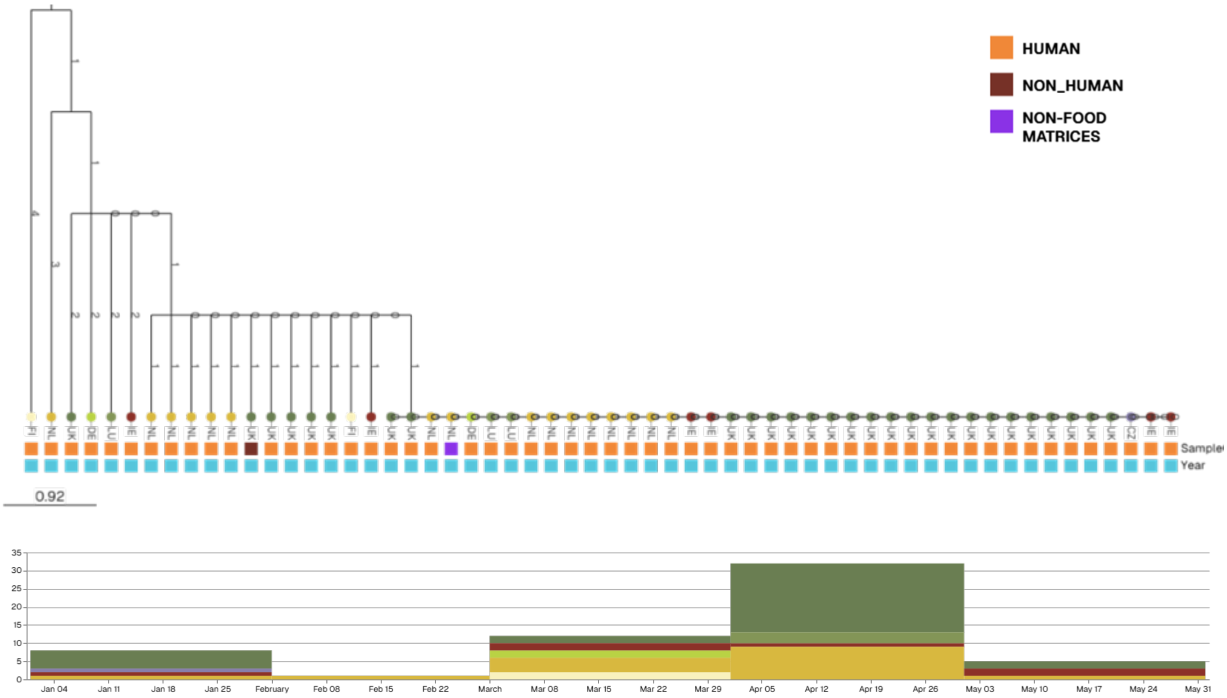

*All isolates are within a 4 AD in a single linkage cluster. There are no other isolates clustering within 80 AD to the outbreak cluster in the ECDC EFSA WGS One Health system.*

## ECDC and EFSA risk assessment for the EU/EEA

Between January and May 2026, 109 confirmed cases of *S.* Bovismorbificans were reported across 10 EU/EEA countries and the United Kingdom, mainly affecting adults and predominantly females. Eighteen cases were hospitalised, and two deaths occurred in Finland among individuals with underlying health conditions, one in a confirmed case and one in a probable case. Epidemiological and microbiological evidence linked the outbreak to the consumption of sprouted seeds, including a recalled brand. Microbiological analysis identified a common outbreak strain (ST377), detected in all affected countries, mainly from domestic cases.

Alfalfa sprouted seeds, and possibly other sprouted seeds, remain the primary food vehicle in this multi-country outbreak, with cases linked to the outbreak. This is based on the consumption of sprouted seeds as reported by the majority of interviewed cases in Finland, the Netherlands, and the United Kingdom and it is further supported by the identification of the outbreak strain (as per EU centralised WGS cluster analysis) in water samples collected during alfalfa sprouted seed harvesting from producers in the Netherlands and the United Kingdom (Northern Ireland), and an epidemiological link between cases in Finland and a Finnish sprouted seed producer.

Other additional sources of infection cannot be excluded, as not all interviewed cases reported consumption of sprouted seeds. However, in epidemiological investigations sprouted seeds are considered stealth vehicles, as their consumption is often difficult to recall. This is largely due to their frequent use as minor ingredients in salads and sandwiches, or as decorative garnishes [1].

Traceability investigations, conducted by the food safety authority in Italy on the alfalfa seeds used for sprouting and associated with the outbreak strain detection in water samples, identified a common seed supplier based in India. These findings suggest this geographical area as the potential origin of the seed contamination with the outbreak strain, which has not previously been reported in the EU/EEA. Bacterial pathogens may survive for long periods on dry seed (e.g. during transport and seed storage) and they can grow during sprouting due to the high humidity and favourable temperatures [5]. Therefore, the role of the environment in seed contamination among growers should be further investigated, and contamination with pathogenic bacteria should be minimised through the application of good agricultural practices (GAPs) [5]. Furthermore, preventing initial contamination during production, and later during storage and distribution of seeds is of the utmost importance since sprouted seeds have the potential to cause large food-borne outbreaks, as seen recently in a multi-serovar *Salmonella* outbreak affecting Europe (509 cases, nine EU/EEA countries, 2023–2025). Operators producing sprouted seed products should implement effective food safety management systems to ensure that only safe products reach the market [1].

The outbreak strain is presumed to have entered Europe in October 2025 via two alfalfa seed consignments imported from India. These consignments were subsequently sorted and distributed (by an Italian supplier) to multiple countries (13 EU/EEA countries, as well as Switzerland and the United Kingdom), consistent with the onset of the first reported cases in January 2026. In May 2026, the food safety authority in Italy requested the withdrawal of alfalfa seeds from the two implicated consignments. In addition, the food safety authorities in the affected countries reported having implemented control measures, including the withdrawal of related sprouted seed products, issuance of public recalls, temporary cessation of sprouted seed production pending investigation, seizure of seeds at production sites, and rejection and destruction of suspected sprouted seed products.

Since these interventions, case notifications have significantly decreased. However, further infections may occur until the source of contamination is fully identified and effectively controlled, particularly because sprouted seeds can be ready-to-eat products, representing a concern for microbial food safety [5].

Based on currently available information, and with no cases reported in June, the risk of *S.* Bovismorbificans infections is assessed as low-to-moderate for people in EU/EEA countries who frequently consume sprouted seeds.

## Recommendations

**Public health authorities** are encouraged to:

- Interview new cases of *S.* Bovismorbificans ST377 to investigate potential links to the consumption of sprouted seeds. To reduce recall bias, the inclusion of pictures of different types of sprouted seed products should be considered during case interviews.
- Sequence human isolates of *S.* Bovismorbificans, if possible, and share them in the ECDC WGS system. For countries with limited or no capacity, ECDC can offer sequencing support.
- Update the EpiPulse event 2026-FWD-00040 should new cases of *S.* Bovismorbificans ST377 be reported.
- Work closely with food safety authorities on national investigations if new *S.* Bovismorbificans ST377 cases are identified.

**Food safety authorities** are encouraged to:

- Share in the EFSA WGS system any sequences of *Salmonella* Bovismorbificans ST377 from food and environmental isolates linked to the present cluster, either microbiologically (serotype or sequence type) or epidemiologically (e.g. suspected food items reported by patients) and share in RASFF the traceability information related to the foods from which these sequences were derived.
- Submit genomic data of *Salmonella* Bovismorbificans isolates from any kind of food, feed, animal or environmental samples to the EFSA WGS System.
- Follow the ISO analytical methods for the microbiological detection of *Salmonella* in dry seeds and full application of the requirements for the sampling of sprouted seeds, as per Chapter 3.3 of Annex I, Commission Regulation (EC)No 2073/2005 of 15 November 2005 on microbiological criteria for foodstuffs [6].

**Consumers** are encouraged to follow proper hygiene practices at home, including rinsing fresh produce and following the manufacturer's instructions when handling and consuming sprouted seeds.

## Source and date of request

ECDC sent a request to EFSA on 8 May 2026 to produce a Joint Rapid Outbreak Assessment (ROA). EFSA accepted the request on 11 May 2026.

## Consulted experts and national contact points

**ECDC experts (in alphabetical order):** Cecilia Jernberg, Elena Portell Buj, Priyanka Nannapaneni, Linda Trönnberg.
**EFSA staff (in alphabetical order):** Laura Cabral, Beatriz Guerra, Valentina Rizzi, Mirko Rossi, Eleonora Sarno, Frank Verdonck.

**Public health experts consulted for data and fact validation:**

**Austria**: Sabine Maritschnik and Christian Kornschober (AGES).
**Belgium**: Dieter Van Cauteren (Epidemiology of Infectious Diseases, Sciensano) and Wesley Mattheus (National Reference Center *Salmonella*, Sciensano).
**Czechia**: Ondřej Daniel, Michaela Špačková and Andrea Mančíková (National Institute of Public Health)
**Denmark**: Gitte Sørensen and Luise Müller (Statens Serum Institut).
**Finland**: Kaisa Jaakkola, Anni Vainio and Heidi Landgren (THL).
**Germany:** Anika Meinen (Department for Infectious Disease Epidemiology; Gastrointestinal Infections, Zoonoses and Tropical Infections Unit; Robert Koch Institute) and Sandra Simon (Department for Infectious Diseases; NRC for *Salmonella* and other bacterial enteric pathogens; Robert Koch Institute).
**Ireland**: Anthony Ortiz, Patricia Garvey (HSE-Health Protection Surveillance Centre), Paul McKeown (HSE-National Health Protection Office) and Martin Cormican (National *Salmonella, Listeria* and *Shigella* Reference Laboratory/Galway University Hospital).
**Luxembourg**: Corinna Ernst, Isabelle Kerrec, Dieta Gashi, Joël Mossong (Direction de la Santé) and Amy Parrish, Catherine Ragimbeau (Laboratoire national de santé).
**Poland**: Małgorzata Milczarek-Lisicka and Tomasz Wołkowicz (NIPH NIH – NRI).
**Spain**: Silvia Herrera-León and Carmen Varela Martínez (Instituto de Salud Carlos III – ISCIII).
**The Netherlands**: Anouk Meijs and Linda Visser (RIVM).
**The United Kingdom**: Hannah Charles, Amy Douglas, Ann Hoban, Thomas Thackray, Gauri Godbole, Amina Ismail-Ahmed, David Greig and Frieda Jorgensen (UKHSA), Patrick McAleavey (Public Health Agency, Northern Ireland), Lynda Browning and Lesley Larkin (Public Health Scotland) and Andrew Nelson (Public Health Wales).

**RASFF contact points consulted:** Belgium, Bulgaria, Croatia, Finland, Germany, Hungary, Ireland, Italy, Lithuania, Luxembourg, the Netherlands, Poland, Portugal, Romania, Slovakia, Slovenia, Spain, Switzerland. United Kingdom (Northern Ireland) (consulted via the European Commission).

**National experts consulted by the RASFF contact points:**

**Finland**: Elina Leinonen and Paula Hietanen (Finnish Food Authority).
**Ireland**: Michelle Minihan, Orla Moore, Karen McCullagh, Christine Mulcahy (The Food Safety Authority of Ireland).
**Italy**: Raffaello Lena, Loredana Iuliano, Claudia A. Grimaldi (Italian Ministry of Health – Office 8 DGISA).
**Hungary**: Krisztina Kovács and Sándor Ádám (National Food Chain Safety Office, Hungary).
**Luxembourg:** Fabienne Clabots and Isabelle Paulus (ALVA, Luxembourg Veterinary and Food Administration)
**Netherlands:** Anouk Bouwhuis, Jacco Janssen, Ife Slegers - Fitz-James (Netherlands Food and Consumer Product Safety Authority).
**Slovenia**: Nadja Skrk, Administration for Food Safety, Veterinary Sector and Plant Protection.
**Spain**: RASFF Team (Spanish Agency for Food Safety and Nutrition, AESAN).
**Switzerland**: RASFF Team (Federal Food Safety and Veterinary Office).
**United Kingdom-Northern Ireland**: Craig Leeman, Jess Cassidy and Joanna Brown (Consumer Protection Division, Food Standards Agency, Northern Ireland).

**Country Officers of the EFSA WGS system:**
Country Officers of the EFSA WGS System were consulted in the Netherlands.
United Kingdom (Northern Ireland) was also consulted.

## Disclaimer

This rapid outbreak assessment was written jointly by the European Centre for Disease Prevention and Control (ECDC) and the European Food Safety Authority (EFSA).

ECDC issued this outbreak assessment document in accordance with Article 20 of Regulation (EU) 2022/2371 on serious cross-border threats to health, Articles 7(1) and 8a of Regulation (EC) No 851/2004 establishing a European Centre for Disease Prevention and Control. EFSA's contribution is based on a mandate from the European Commission requesting EFSA to provide scientific assistance from EFSA in the investigation of multinational food-borne outbreaks (Ares (2013) 2576387, Mandate M-2013-0119, 4 July 2013) in accordance with Article 31 of Regulation (EC) No 178/2002 of the European Parliament and of the Council of 28 January 2002, laying down the general principles and requirements of food law, establishing the European Food Safety Authority and laying down procedures in matters of food safety.

The specific purpose of an ECDC-EFSA rapid outbreak assessment is to present an analysis of a cross-border food-borne threat to health and to provide science-based recommendations and options for response. The responsibility for the choice of which options to pursue and which actions to take at national level, following ECDC and EFSA's recommendations, lies with EU/EEA countries.

All data published in this rapid outbreak assessment are data collected from EU and/or EEA countries concerned by the outbreak until the date this assessment was produced. Maps and figures published do not represent statements from ECDC or EFSA on the legal or border status of the countries and territories shown but constitute the information on which this rapid outbreak assessment is based.

## Annex 1. Microbiological and environmental investigations of food and control measures

### United Kingdom

On 20 April 2026, the European Commission issued a RASFF notification (Alert Notification 2026.3378) on behalf of the United Kingdom (Northern Ireland) to report on the detection of *S.* Bovismorbificans in a sample of water collected during an own check control at the premises of sprouted seed Producer A located in the United Kingdom (Northern Ireland). Further national investigation confirmed a microbiological link between the water irrigation isolate and human isolates from eight cases of infection (January – March 2026) initially investigated. The water sample (irrigation water from sprouted seeds, planted on 12 March 2026) was collected on 14 March 2026.

The food authority reported that the sprouted seed products associated with the positive result from the irrigation water expired on 1 April 2026 (2026.3378, *fup3*, 2026.3378). As a control measure, on 22 April 2026 the food safety authority in the United Kingdom (Northern Ireland) issued a public warning to recall the sprouted seed products grown at sprouted seed Producer A (with expiry dates up to beginning of May) (*fup6*, 2026.3378). In addition, the food authority also reported that Producer A had ceased production of the sprouted seeds while investigations were ongoing. The products had been distributed to business operators in the United Kingdom and Ireland (*fup6*, 2026.3378).

At the time that the irrigation water tested positive water, different seeds had been used for sprouting, namely alfalfa seeds from Batch C, clover seeds from Batch D, broccoli seeds from Batch E, and radish seeds from Batch F (2026.3378). These seeds had been supplied by the Italian seed Supplier A.

### Finland

On 21 April 2026 the food safety authority in Finland shared the findings of the ongoing national outbreak investigation (*fup1*, 2026.3378) in RASFF. One Finnish producer of sprouted seeds (Producer B) had been linked epidemiologically with some cases of *S.* Bovismorbificans infection. During the outbreak period, different batches of seeds were used on the premises of Producer B (alfalfa seeds from Batch AJ, green lentil seeds from Batch CC, brown lentil seeds from Batch CA, radish seeds Batch G, black onion seeds from Batch AI, and mung beans). The Producer B had received the alfalfa seeds Batch AJ and black onion seeds Batch AI on 19 October 2025, and the alfalfa seeds Batch CG, the green lentil seeds Batch CC, the brown lentil seeds Batch CA, and the radish seeds Batch G on 14 June 2023 from the Italian seed Supplier A. The mung beans were submitted by a different supplier to Supplier A (*fup1*, *fup12*, 2026.3378).

The Producer B stopped using seeds from Batch AJ in late April and the products still on the market were withdrawn (*fup50*, 2026.3378). In late May, sprouted seed products from alfalfa seeds Batch BW were recalled (*fup50*, 2026.3378).

### Ireland

On 22 April 2026, the food safety authority in Ireland (Food Safety Authority of Ireland) issued a public warning and initiated a recall of implicated batches of sprouted seeds produced by the sprouted seed Producer A, located in the United Kingdom (Northern Ireland) following the identification of three confirmed cases in Ireland. The products had been distributed to retailers in the Republic of Ireland.

On 23 April 2026, the authority made a request in RASFF for the full distribution list of food business operators in Ireland supplied with alfalfa, clover, broccoli and radish seeds from the Italian seed Supplier A (*fup4*, 2026.3378).

### Netherlands

On 21 May 2026, the food safety authority in the Netherlands reported in RASFF that an official control had been carried out at the premises of sprouted seed Producer F that had received various seeds in different batches from the Italian seed Supplier A. An own check control sample of rinsing water from a harvest of sprouted alfalfa seeds (made with seeds supplied by the Italian seed Supplier A) tested positive for *S.* Bovismorbificans on 14 February 2026. The harvest of sprouted seeds associated with the positive result (positive rinsing water sample) has been destroyed and was not traded. This harvest was obtained with alfalfa seeds Batch AS (*fup43*, *fup49*, 2026.3378) and was not marketed, but rejected and destroyed. The authority reported that at Producer F each harvest is tested for *Salmonella* before release. Therefore, the other harvests of sprouted seeds previously obtained with seeds from Batch AS tested negative (i.e. *Salmonella* was not detected in additional rinsing water tests) before being distributed to food business operators in Belgium, Germany, Spain, and within the Netherlands (*fup43*, *fup49*, *fup55*, 2026.3378). In addition, the authority shared information on the distribution of the other sprouted seed products obtained with the seeds received in Batch AL (delivered within the Netherlands), Batch AM (delivered to Belgium, Germany, Spain, and within the Netherlands), and Batch AN (delivered within the Netherlands) (*fup49*, *fup55*, 2026.3378).

### Italy

On 24 April 2026, the food safety authority in Italy shared (*fup5*, 2026.3378) the outcome of the food traceability investigations related to the suspected batches of alfalfa seeds, red clover seeds, broccoli seeds, and radish seeds identified in the outbreak investigations in the United Kingdom (Northern Ireland), Finland and the Netherlands, in RASFF, as requested (RASFF 2026.3378). The results of the traceback investigations related to the black onion seeds, and brown and green lentil seeds were also shared. All these seeds were traded in Europe and outside of Europe by the Italian seed Supplier A.

#### *Alfalfa seeds*

The Italian seed Supplier A delivered the alfalfa seeds Batch C (no longer in stock at the time of the notification) to the sprouted seeds Producer A located in the United Kingdom (Northern Ireland). Batch C derived from Batch AK. In addition, the Italian seed Supplier A delivered the alfalfa seeds Batch AJ to the sprouted seed Producer B located in Finland on 19 October 2025. Batch AJ derived from Batch AK.

Batch AK was renamed at the Italian seed Supplier A and further batches were generated that were marketed in the following European countries: the Netherlands (delivered to the sprouted seed Producer F as Batch AL, Batch AM, and Batch AN); Bulgaria (delivered to the sprouted seed Producer E) as Batch AO; United Kingdom (Northern Ireland) (delivered to the sprouted seed Producer A as Batch C and Batch AP; Switzerland (delivered to the sprouted seed Producer D and sprouted seed Producer H as Batch AQ and Batch AR, respectively). Deliveries were also made outside of Europe. A remaining part of Batch AK is stored at the Italian seed Supplier A (*fup19*, 2026.3378).

Batch AK was produced from the alfalfa seeds Batch A delivered to the Italian seed Supplier A by the Italian seed Supplier B on 23 October 2025 (*fup5*, 2026.3378). Part of this batch was in storage and was seized during the official health control by the authority (*fup15*, 2026.3378). At the Italian seed Supplier B, the batch was tested during an own check control in November 2025. *Salmonella* was not detected (*fup10*, 2026.3378). On 24 April 2026, the authority carried out an official control at the Italian seed Supplier B regarding the traceability of alfalfa seeds Batch A. The alfalfa seeds Batch A had been acquired by the Italian seed Supplier B from the Indian Supplier C as alfalfa seeds Batch A which had arrived in Italy from India on 10 August 2025.

Moreover, the Italian seed Supplier A delivered the alfalfa seeds Batch CG to the sprouted seed Producer B on 18 July 2023. Batch CG derived from Batch CH. The Italian seed Supplier A sourced Batch CH from the Italian Grower F (*fup48*, 2026.3378).

Upon request from the authority in the United Kingdom, the food safety authority in Italy asked the Italian seed Supplier A to provide the complete list of batches of seeds delivered to food business operators in the United Kingdom between October 2025 and April 2026, and to food business operators in the United Kingdom (Northern Ireland) between November 2025 and April 2026 (*fup7*, 2026.3378).

The Italian seed Supplier A delivered the alfalfa seeds Batch AS to the sprouted seed Producer F located in the Netherlands on 13 November 2025.

Batch AS derived from Batch AT. Batch AT originated from the alfalfa seeds Batch B delivered on 23 October 2025 to the Italian seed Supplier A by the Italian seed Supplier B. This batch had been acquired from the Indian Supplier C as above.

Batch AT was renamed at the Italian seed Supplier A and generated further batches that that were marketed in the following European countries: Hungary (delivered to the seed Seller C as Batch AU, Batch AV, Batch AW, Batch AX, Batch AY); Spain (delivered to the sprouted seed Producer I as Batch BA); the Netherlands (delivered to the sprouted seeds Producer F as Batch BB; Batch AS; Batch CL; and Batch BC); United Kingdom (Northern Ireland) (delivered to the sprouted seed Producer A as Batch BD); Lithuania (delivered to the sprouted seed Producer P) as Batch BE and Batch BF; Poland (delivered to the seed Seller E as Batch BG and Batch BH; delivered to seed Seller B as Batch BI and as Batch BJ; delivered to the seed Seller K as Batch CE; delivered to the sprouted seed Producer N as Batch CM); Switzerland (delivered to the sprouted seed Producer D as Batch BK and delivered to the seed Seller F as Batch BL); Slovenia (delivered to the seed Seller G as Batch BM and Batch BN); United Kingdom (delivered to the seed Seller A as Batch BO and Batch CJ; delivered to the seed Seller H as Batch BP and Batch BQ); Romania (delivered to the sprouted seed Producer J as Batch BR); Portugal (delivered to the sprouted seed Producer K as Batch BS and Batch BT); Italy (delivered to seed Seller I as Batch BU, and delivered to the seed Seller J as Batch BV and Batch BX); Slovakia (delivered to the sprouted seed Producer L as Batch BY); Finland (Producer B as Batch BW); Bulgaria (sprouted seed Producer E as Batch BZ. Deliveries were also made outside of Europe: Singapore. A remaining part of Batch AT is stored at the Italian seed Supplier A (*fup22*, 2026.3378).

#### *Red clover seeds*

The Italian seed Supplier A delivered the red clover seeds from Batch D (no longer in stock at the time of the notification) to the sprouted seed Producer A located in the United Kingdom (Northern Ireland).

Batch D was produced from red clover seeds Batch H delivered on 06 March 2025 to the Italian seed Supplier A by the Italian seed Supplier B (*fup5*, 2026.3378).

Batch H was renamed at the Italian seed Supplier A and became Batch I and was distributed to the United Kingdom (Northern Ireland) (sprouted seed Producer A) as Batch D and Batch J and outside of Europe (*fup24*, 2026.3378). At the Italian seed Supplier B, the batch tested negative for *Salmonella* during an own check control on 17 March 2025 (date of report issued) (*fup10*, 2026.3378). On 24 April 2026, the food safety authority carried out an official control on the premises of the Italian seed Supplier B regarding the traceability of red clover seeds Batch H. This batch had been acquired from the US Supplier D as red clover seeds Batch H and arrived in Italy on 25 December 2024.

#### *Broccoli seeds*

The Italian seed Supplier A delivered the broccoli seeds Batch E (no longer in stock at the time of the notification) to the sprouted seed Producer A located in the United Kingdom (Northern Ireland). Batch E was produced from broccoli seeds Batch K delivered to the Italian seed Supplier A from the Italian Grower A on 17 June 2024.

Batch K was renamed at the Italian seed Supplier A and further batches were then generated that were marketed in the following European countries: United Kingdom (delivered to the seed Seller A as Batch L); Poland (delivered to the sprouted seed Producer C as Batch M, Batch N, Batch O; delivered to the seed Seller B as Batch P and Batch Q); United Kingdom (Northern Ireland) (delivered to the sprouted seed Producer A as Batch R, Batch S, Batch E, and Batch T); Spain (delivered to the sprouted seed Producer I as Batch U and Batch V); Switzerland (delivered to the sprouted seed Producer D as Batch W), Bulgaria (delivered to the sprouted seed Producer E as Batch X). Deliveries were also made outside of Europe: Taiwan and Japan (*fup26*, 2026.3378).

#### *Radish seeds*

The Italian seed Supplier A delivered the radish seeds Batch F (no longer in stock at the time of the notification) to the sprouted seed Producer A located in the United Kingdom (Northern Ireland). Batch F was produced from radish seeds Batch Y delivered to the Italian seed Supplier A by the Italian Grower B on 08 July 2025 (*fup5*, 2026.3378). During the official control carried out at the Italian Grower B, the company reported that it did not irrigate the fields where seeds are grown, but fertilised them using triple superphosphate and urea (*fup17*, 2026.3378).

Batch Y generated further batches that that were marketed in the following European countries: Poland (delivered to sprouted seed Producer C as Batch Z and Batch AA; and delivered to the seed Seller B as Batch AB); Hungary (delivered to the seed Seller C as Batch AC and Batch AD); the Netherlands (delivered to the sprouted seed Producer F as Batch CK); United Kingdom (Northern Ireland) (delivered to the sprouted seed Producer A as Batch F and Batch AE); Bulgaria (delivered to the sprouted seed Producer E as Batch AF; Italy (delivered to the seed Seller D as Batch AG); Switzerland (delivered to the sprouted seed Producer G as Batch AH). Deliveries were also made outside of Europe: Japan (*fup25*, 2026.3378).

With regard to the radish seeds Batch G, the Italian seed Supplier A delivered Batch G to the sprouted seed Producer B located in Finland. Batch G belongs to Batch CI. The Italian seed Supplier A sourced Batch CI from the Italian Grower G (*fup48*, 2026.3378).

#### *Black onions*

The Italian seed Supplier A delivered the black onion seeds Batch AI (no longer in stock at the time of the notification) to the sprouted seed Producer B located in Finland on 19 October 2025. Batch AI was produced from black onion seeds delivered to the Italian seed Supplier A from the Italian Grower C on 29 August 2023 (*fup5*, *fup21*, 2026.3378).

#### *Brown lentil seeds* and green lentil seeds

The Italian seed Supplier A delivered the brown lentil seeds Batch CA to the sprouted seed Producer B. The Italian seed Supplier A purchased the brown lentil seeds from this batch on 28 June 2022 from the Italian Grower D and assigned a new batch number Batch CB upon receipt that was renamed as Batch CA for distribution to the Producer B (*fup29*, 2026.3378).

The Italian seed Supplier A delivered the green lentil seeds from Batch CC to the Producer B. The Italian seed Supplier A purchased the green lentil seeds on 6 July 2022 from the Italian Grower E and upon receipt assigned the new batch number Batch CD that was renamed as Batch CC and further distributed to the Producer B (*fup29*, 2026.3378).

#### *Control measures*

On 19 May 2026, the food safety authority in Italy reported that the Italian seed Supplier A had been asked to implement withdrawal procedures for the alfalfa seeds in Batch A which was further designated as Batch AK and distributed with different batch codes to the countries (*fup19*, 2026.3378) concerned (*fup41*, 2026.3378).

On 22 May 2026, the food safety authority reported that the withdrawal procedures had been extended to alfalfa seeds Batch B further designated as Batch AT and distributed with different batch codes to the countries (*fup22*, 2026.3378) concerned (*fup45*, 2026.3378). In addition, the authority reported that the sampling of the alfalfa seeds (Batch B and Batch A) intended for microbiological testing was ongoing at the Italian seed Supplier A while the authority considered the clover seeds, the radish seeds, and the broccoli seeds as compliant (*fup45*, 2026.3378).

### Luxembourg

On 8 May 2026, the food safety authority reported the outcome of the national food investigation carried out on the mix of various sprouted seeds that (potentially) could have been the cause of the infection in RASFF. The care home, where one case had been exposed, had received a delivery from the Luxembourg Wholesaler A on 4 April 2026. The Luxembourg Wholesaler A sourced the products (a mix of various sprouted seeds produced by the Dutch sprouted seed Producer O) from the Dutch Wholesaler B. The authority reported that the exposure was only suspected and therefore it has not been confirmed that the patient consumed these products. However, the authority reported in RASFF that a cross-contamination event could not be ruled out (*fup23*, 2026.3378). Furthermore, the authority informed that eight different sorts of sprouted seed products were sampled at the Luxembourg Wholesaler A and analysed. Four out of eight products originated from the same Dutch sprouted seed Producer O. All samples tested negative (*fup61*, 2026.3378).

### Slovenia and Croatia

On 8 May 2026, the food safety authority in Slovenia reported the outcome of its food investigations in RASFF, noting that alfalfa seeds Batch BN had been distributed to a sprouted seed Producer M on 23 January 2026 (*fup27*, 2026.3378). On 11 May 2026, the food safety authority in Croatia reported that the sprouted seed Producer M had not used the batch received (*fup28*, 2026.3378)

### Lithuania

On 13 May 2026, the food safety authority reported the outcome of the official control carried out at the sprouted seed Producer P that had received the alfalfa seeds from the Italian seed Supplier A to RASFF. It was ordered that the seeds should be prohibited from being marketed, and that they should be withdrawn from the market and destroyed (*fup33*, 2026.3378).

### Bulgaria

On 13 May 2026, the food safety authority clarified in RASFF that the seeds received in Bulgaria were not intended for human consumption, but as seeds for sowing. The entire quantity received will be destroyed (*fup34*, 2026.3378) (*fup40*, 2026.3378).

### Poland

On 13 May 2026, the food safety authority reported that the seed Seller B (a company that trades seeds), listed as recipient of two batches of alfalfa seeds and one batch of radish seeds from the Italian seed Supplier A, had not yet been informed by the supplier of the recall. The company confirmed the delivery of alfalfa seeds Batch BI dated 16 January 2026 and one delivery of radish seeds Batch AB dated 6 November 2025. The second delivery of alfalfa seeds Batch BJ dated 23 April 2026 had not yet arrived. The seeds not already sold were blocked (*fup35*, 2026.3378). On 22 May 2026, the authority reported having been notified by the Italian seed Supplier A of the withdrawal procedures (Batch BI) and having proceeded with implementation (*fup47*, 2026.3378).

With regard to the sprouted seed Producer N the food safety authority reported that the company confirmed the receipt of alfalfa seeds Batch BJ from the Italian seed Supplier A. The seeds in question were seized, not used for sprout production, and returned to the supplier (*fup36*, 2026.3378).

With regard to the sprouted seed Producer C, the food safety authority reported that the company received radish seeds Batch AA (awaiting production) from the Italian seed Supplier A on 9 March 2026, and on 9 March 2026 broccoli seeds Batch O (entirely used for sprout production). On 30 April 2025 it had received broccoli seeds Batch N (entirely used for sprout production); on 31 March 2025 broccoli seeds Batch M (all used for sprout production), and on 2 November 2025 radish seeds Batch Z (all used for sprout production) (*fup37*, 2026.3378).

With regard to the seed Seller L the food safety authority reported that the company had suspended the sprouting of broccoli seeds Batch CF received from the Italian seed Supplier A (*fup38*, 2026.3378).

### Hungary

On 15 May 2026, the food safety authority reported that the seed Seller C which had received the seeds from the Italian seed Supplier A does not produce sprouts and operates exclusively as a seed packaging company, packaging seeds intended for home sprouting by consumers. The food authority carried out an inspection at the plant and seized the remainder of alfalfa seed Batch AT. With regard to radish seeds Batch Y, these had already been distributed. The company was ordered to carry out the withdrawal from the market and recall of the alfalfa and radish seed batches. The company was also instructed to ensure that any remaining stock batches, once identified, were stored separately and clearly marked (official detention), pending further measures. The food batches concerned must be stored separately from other products in a clearly visible manner. The authority ordered the withdrawal of the products from the market and the recall of products that had already reached consumers. It prohibited the placement of any further products on the market pending additional official measures (*fup39*, 2026.3378).

### Switzerland

On 21 May 2026, the food safety authority reported that the seeds delivered to Switzerland had been seized and destroyed (*fup44*, 2026.3378). The authority also reported that according to the Federal Office of Public Health, there had been no increase in the number of human cases of *S.* Bovismorbificans recorded in 2026.

### Romania

On 22 May 2026, the food safety authority reported in RASFF that a sample from alfalfa sprouts (48 hours after the start of the germination process) obtained with seeds Batch BR had been collected at the sprouted seed Producer J. The sample tested negative for *Salmonella* on 8 May 2026. However, the authority reported in RASFF that it had ordered the destruction of the batch, given that the expiry date for Batch BR had passed (*fup46*, 2026.3378).

## Annex 2. Background information on *S.* Bovismorbificans in food

### Food-borne outbreaks caused by *S.* Bovismorbificans in sprouted seed products

Country specific data on food-borne outbreaks associated with *Salmonella* in sprout products are reported to EFSA by countries in accordance with the Zoonoses Directive 2003/99/EC.

Overall, four strong-evidence foodborne outbreaks caused by *Salmonella* Bovismorbificans in sprouted seed products have been reported (as reported to EFSA up to 2025), with 118 human cases, 16 hospitalisations and no deaths. The outbreaks were reported by Finland (2009), Germany (2014), Switzerland (2014) and Estonia (2009).

### Occurrence of *S.* Bovismorbificans in food

This section summarises country-specific data on the occurrence of *Salmonella* for the matrix seeds and sprouted seeds, representing the main food categories involved in this assessment, as reported to EFSA up to 2025 by the countries in accordance with the Zoonoses Directive 2003/99/EC.

For the food categories 'Seeds and sprouted seeds', Ireland (2010) and Germany (2014) reported five 'total units positive' out of 440 'total units tested' for *Salmonella*.
